# Differential Effects of Landiolol in Patients with Atrial Fibrillation and Atrial Tachycardia

**DOI:** 10.3390/medicina60111782

**Published:** 2024-10-31

**Authors:** Kengo Ayabe, Tomoyoshi Komiyama, Hiroyuki Takekawa, Honsa Kang, Yasuaki Tsumagari, Miwa Ito, Keiichi Ashikaga, Yoshisato Shibata

**Affiliations:** 1 Cardiovascular Center, Miyazaki Medical Association Hospital, Miyazaki 880-2102, Japan; fun3473@gmail.com (H.T.); yasuaki-t@cure.or.jp (Y.T.); ito-m@cure.or.jp (M.I.); ashikaga@cure.or.jp (K.A.); yshibata@cure.or.jp (Y.S.); 2 Department of Clinical Pharmacology, Tokai University School of Medicine, Isehara 259-1193, Japan; 3 Fukuoka City Hospital Cardiology Department, Fukuoka 812-0046, Japan; f030eb@gmail.com

**Keywords:** landiolol, HFpEF, HFmrEF, HFrEF, β-blocker

## Abstract

Landiolol, an ultra-short-acting β1-selective blocker, is more effective in controlling heart rate compared with digoxin in patients with atrial tachyarrhythmias and left ventricular dysfunction. However, the effect of atrial tachyarrhythmia type on the effectiveness of landiolol remains unknown. Hence, this study aimed to evaluate the effectiveness of landiolol in patients with atrial fibrillation (AF) and atrial tachycardia (AT), not limited to those with heart failure with a reduced ejection fraction. To this end, we evaluated the efficacy and safety of landiolol in managing tachycardiac AF and tachycardiac atrial flutter/AT in 44 patients with reduced left ventricular function. We found that while landiolol was effective in managing patients with AF and heart failure with a preserved or mid-range ejection fraction, however, it might be more challenging to control heart rate in patients with AT using a similar dosage of landiolol.

## 1. Introduction

Although we found that landiolol was effective in managing patients with heart failure (HF) complicated by atrial fibrillation (AF), achieving heart rate (HR) control via landiolol treatment may be more challenging in patients with atrial tachycardia (AT) compared with AF. Landiolol, an ultra-short-acting and relatively novel β-blocker, is widely used to control HR in patients with AF and AT [1]. As a super-selective β1-adrenergic antagonist, landiolol inhibits the positive chronotropic effects of the catecholamines adrenaline and noradrenaline on the heart (where β1 receptors are primarily located) while effectively reducing HR, minimally affecting blood pressure and myocardial contractility [2,3]. Such β-blockers play an important role in reducing mortality in patients with coronary artery disease and HF [4,5]. There are several types of β-blockers, such as carvedilol, bisoprolol, and metoprolol, available on the market. Although the CIBIS trial regarding bisoprolol did not demonstrate a significant difference in mortality among patients with HF, other studies have proven the effectiveness of β-blockers in reducing mortality among patients with HF [6,7]. Landiolol, an ultra-short-acting and relatively novel β-blocker, is widely used to control HR in patients with AF and AT [1]. In Europe. landiolol is used for a short-term reduction in ventricular rate in patients with supraventricular tachycardia, including AF and AT, not limited to HF with reduced ejection fraction, especially when short-term control of ventricular rate with a short-acting agent is desirable [8]. In Japan, landiolol has been approved for the treatment of AF and atrial flutter in patients with impaired left ventricular function since 2013 [9]. Since AF or AT with a rapid ventricular response can cause syncope, chest pain, and dyspnea because of reduced stroke volume, increased cardiac oxygen demand, and tachycardia-induced cardiomyopathy, appropriate HR control using β-blockers is critical [10]. There are several research works on the effectiveness of landiolol. However, these studies have usually focused on patients with HF with poor left ventricular function [9]. Although this drug is intended for use in patients with a low left ventricular ejection fraction (LVEF), it is sometimes administered to patients with HF with a preserved EF (HFpEF) or a mid-range EF (HF with a mildly reduced EF: HFmrEF). HFpEF, HFmrEF, and HFrEF are defined as follows: HFpEF with an LVEF of 50% or higher, HFmrEF with an LVEF of 40–50%, and HFrEF with an LVEF of 40% or less [11]. It has been reported that landiolol may be ineffective in managing AT with HFrEF. However, the effectiveness of landiolol in managing patients with AF or AT that have HFmrEF or HFpEF remains unknown [12]. The cost-effectiveness of landiolol in preventing the occurrence of AF in patients in the postoperative stage has already been proven. However, its efficacy in managing AT remains to be determined [13]. If landiolol proves less effective than anticipated in patients with AF or AT and HFpEF or HFmrEF, the cost-effectiveness of its use may need to be reconsidered. Thus, this study aimed to evaluate the varying effects of landiolol on patients with AF and AT, not limited to HFrEF. We also report a case of a patient with AT who received landiolol.

## 2. Materials and Methods

### 2.1. Data Collection

This single-center, retrospective study involved a medical chart review. Laboratory data and cardiac studies, including echocardiograms, were collected and analyzed. From January 2019 to December 2020, the data of patients admitted to the Department of Cardiology at Miyazaki Medical Association Hospital who received landiolol treatment for supraventricular arrhythmias with a rapid ventricular response, such as AF and AT, were retrospectively analyzed.

All data were recorded in the form of electronic records at our institute; their accuracy and validity were verified because most of the vital signs, including HR, blood pressure, and oxygen saturation, were recorded automatically and transmitted to the electronic record system at our institute.

**Figure 1:**
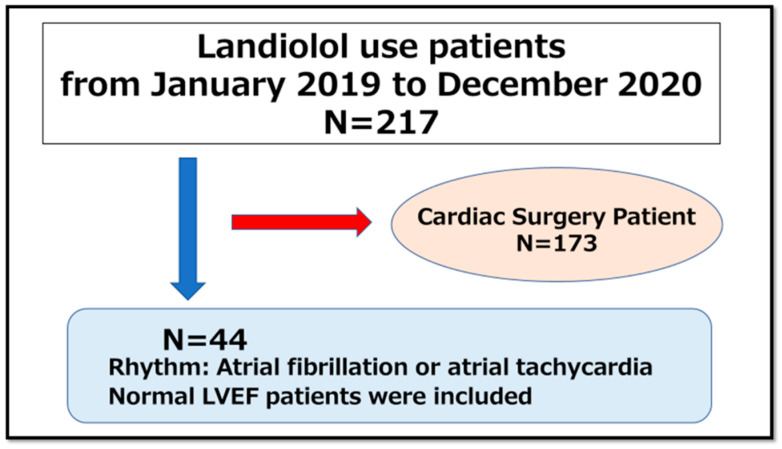
Selection of study participants.

Overall, 217 patients received landiolol during the study period. However, 173 patients were excluded from this study for the following reasons: regular administration of landiolol, such as in intraoperative situations, regardless of heart problems, and the reasons for landiolol use not being clearly documented in a patient’s medical charts. Patients who underwent cardiac surgery were also excluded because these are invasive procedures and involve multiple factors that could affect HR in general. Ultimately, 44 patients were included in the study (Figure 1).

### 2.2. Patient Exclusion Criteria

Patients who underwent cardiac or noncardiac surgery were excluded from this study. There were two reasons for this decision. First, patients who have undergone cardiac surgery often present with atrial or ventricular arrhythmias, and our anesthesiologists administered landiolol intraoperatively regularly. Second, surgery is invasive, during which numerous factors can influence HR and rhythm, such as pain, bleeding, and dehydration.

### 2.3. Definition of the Effectiveness of Landiolol

Effective treatment was defined as achieving a reduction in HR to below 110 bpm and a 20% reduction within 12 h following landiolol administration. The dosage of landiolol and decisions regarding its discontinuation were made at the discretion of each physician. Vital signs, including blood pressure and HR, were continuously recorded using an arterial line and a telemetry monitor.

### 2.4. Statistical Analyses

All statistical analyses were performed using EZR version 1.68 statistical software (Saitama Medical Center, Jichi Medical University, Saitama, Japan), which is a graphical user interface for R version 4.20 (The R Foundation for Statistical Computing, Vienna, Austria). More precisely, it is a modified version of the R commander designed to add statistical functions frequently used in biostatistics [14]. Data were analyzed using the log rank and the Mann–Whitney *U* tests.

## 3. Results

### 3.1. Patient Selection and Baseline Characteristics

Forty-four patients were evaluated; the results are presented in Table 1. Their LVEF and brain natriuretic peptide (BNP) levels were 42.7% ± 16.5% and 664 ± 440 pg/mL, respectively. The percentages of landiolol responders and non-responders were 54.5% (*n* = 24) and 45.5% (*n* = 20), respectively. The number of patients with HFpEF, HFmrEF, and HFrEF was 14, 10, and 20, respectively. Four patients underwent percutaneous coronary intervention for ischemic heart disease during admission, and none of them experienced any complications due to the intervention. No patients underwent catheter ablation during admission. No patient received beta-adrenergic medications such as dobutamine at the same time as landiolol administration. The characteristics of these individuals are summarized in Table 2. The LVEFs for responders and non-responders were 42.0 ± 17.1 and 43.6 ± 16.1 (*p* = 0.78), respectively. Glycated hemoglobin (HbA1c) was the only factor that significantly differed between the two groups; no significant difference was noted after the logistic regression analysis (odds ratio, 1.15; 95% confidence interval, 0.29–1.15; *p* = 0.11).

### 3.2. Patient Characteristics and Different Reactions to Landiolol Between AT and AF

The patient characteristics of those with AT and AF are shown in Table 3. There were no significant differences in age, hemoglobin, BNP, or LVEF between the groups. Additionally, there was no significant difference in the dosage of landiolol between the AT and AF patients. Seven patients presented with AT; however, none responded to landiolol. Although the HR in the patients with AF decreased from 137 to 107 bpm, the HR of those with AT only decreased from 143 bpm to 140 bpm. Thus, landiolol was ineffective in managing AT compared with AF. No adverse events, such as bradycardia (HR ≤ 40 bpm) or hypotension (SBP < 80 mmHg), were observed.

No AT patient returned to sinus rhythm within the observation period, defined as within 12 h following landiolol administration. One patient required cardioversion because of hemodynamic instability and eventually underwent catheter ablation because of recurrent AT. The rhythm of four patients converted from AT into AF, and two patients returned to sinus rhythm 24 h after the initiation of landiolol administration.

## 4. Discussion

This study demonstrated the effectiveness of landiolol in managing AF and AT in patients not only with HFrEF but also HFmrEF and HFpEF. We found that HR in patients with AT may be difficult to control compared with that in individuals with AF by administering a similar dosage of landiolol. This could be attributed to several factors. First, the re-entry mechanism usually contributes to AT maintenance. AF is usually caused by irregular firing from the pulmonary veins (PVs) and other sites known as non-PV foci [15]. Landiolol showed negative chronotropic effects and could potentially help to terminate AF and restore sinus rhythm [16,17]. Considering previously reported related findings, landiolol might not be as effective in extending the cycle length of AT; however, it can be effective in suppressing firing from the PVs and other sites. Additionally, landiolol may be pharmacologically weaker compared with other β-blockers in its ability to control the conduction rate of the atrioventricular node. These factors were considered when analyzing the different effects of landiolol on AT and AF. Landiolol is effective in patients with AF, as demonstrated in previous studies [1]. However, we found that the ratio of successful treatment with landiolol in patients with AF was 54%, which was lower than anticipated. Although the dosage of landiolol in our study might reflect real-world administration practices, the relatively lower dosage could potentially explain the insufficient treatment success rate. It is also noted that there were no adverse events with the administration of landiolol in this study. This finding is compatible with the previously reported data [18].

As described in this case, effective treatment of AT may be catheter ablation rather than simple HR control with β-blockers. In a recent study, spontaneous conversion to sinus rhythm in patients with AF varied between 9% and 83% [19]. Levy et al. conducted a meta-analysis and reported that the efficacy of landiolol in controlling HR and facilitating conversion to sinus rhythm in non-surgical patients was 75.7% [20]. There is a paucity of reports on the spontaneous conversion to sinus rhythm in patients with AT. In our study, the sinus conversion rate in patients with AF was 44.1%, whereas in patients with AT, it was 0% following landiolol administration. It has also been reported that amiodarone could play an important role in converting AF into sinus rhythm. However, none of our patients received amiodarone during landiolol administration [21]. Numerous reports favor catheter ablation of AT [22]. A recently published meta-analysis found that catheter ablation is superior to medical therapy for reducing mortality and cardiovascular rehospitalizations in AF patients, with high certainty [23]. It is also reported that atrioventricular node ablation and device implantation, such as cardiac resynchronization therapy, are effective in treating refractory AF and AT patients [24]. It is important to note that conversion to sinus rhythm from AF or AT is a crucial aspect of treatment. We have also noted that it is essential to acknowledge that catheter ablation procedures are associated with several fatal complications, such as cardiac tamponade and transesophageal fistula cerebral stroke. Notably, these procedures may sometimes require general anesthesia [25].

When physicians administer landiolol to patients with AT and AF, they still consider other treatment options given that landiolol is only partially effective. Furthermore, the cost-effectiveness of landiolol is considered. Landiolol is very expensive compared with other β-blockers. Therefore, continuing landiolol treatment in the absence of substantial effects might not be economical. In this study, determining the precise duration of landiolol administration was very difficult due to the system used for medical records. Landiolol was either discontinued or switched for another inexpensive β-blocker at each physician’s discretion. In the future, we intend to investigate the cost-effectiveness of landiolol further.

## 5. Limitations

This study has some limitations. First, it included a small number of patients and was performed retrospectively at a single center. Therefore, this study can be considered a pilot study to evaluate the effectiveness of landiolol in AT. We plan to increase the number of study participants to strengthen our findings in the future. Second, this study did not consider some important factors that could influence HR, such as other medications and mechanical ventilation. Because this was a retrospective study conducted in the real-world setting of a busy intensive care unit at Miyazaki Medical Association Hospital, certain medications, such as verapamil, a short-acting calcium channel blocker, were administered to some patients at the physician’s discretion. The patients who received these medications did not respond to either landiolol or other treatments. Therefore, we determined that the effect of concomitant medication use was minimal. Additionally, since no patient was intubated or extubated during the observation period, we considered the impact of mechanical ventilation to be minimal as well. In the future, we will consider the factors mentioned above to improve the quality of our study. Third, the landiolol dose used was at the discretion of each physician. This study was retrospective; the dose of landiolol was not increased to the maximum in all patients. Landiolol may be ineffective in patients with AT with HFmrEF and HFpEF, as previous studies have shown different responses to landiolol in AF and AT. The dosage of landiolol in this study (up to 10 μg/kg/min) was relatively low compared to that recommended in the drug information. Increasing the dose of landiolol may lead to a higher number of responders. In several previous studies, the maximum dosage has rarely been administered to patients, except in the J-land study, which was a prominent clinical trial conducted in Japan [1,9]. Although the effect of landiolol depends on the dosage, the average dosage in several previous studies was 3–5 μg/kg/min, which is similar to the dosage in our study. Therefore, we can suggest that our findings may be compatible with those in other studies on landiolol, even though the dosage in our study did not reach the maximum recommended level [9]. Furthermore, the mechanism of AT was not thoroughly investigated in this study. Therefore, clarifying the reasons for the different effects of landiolol between AF and AT might be challenging.

## 6. Conclusions

In conclusion, our findings suggest that landiolol was effective in the management of patients with AF and HFmrEF, as it controlled HR without severe adverse events and contributed to sinus rhythm recovery. However, achieving appropriate control of HR by administering landiolol to patients with AT may pose greater challenges compared with those with AF. Landiolol may be ineffective for AT cycle length shortening and did not contribute to sinus recovery. AT patients may require additional procedures, such as cardioversion and catheter ablation, to return to sinus rhythm.

## Figures and Tables

**Table 1:** Clinical characteristics of this study’s patients.

Patient Characteristics (N = 44)	
Age (years)	78.6 ± 11.1
Male patients (n, %)	27 (61.3)
Weight (kg)	56.7 ± 12.8
Hb (g/dL)	12.3 ± 2.9
BNP (pg/mL)	664 ± 440
HbA1c (%)	6.2 ± 1.1
Creatinine (mg/dL)	1.4 ± 1.1
AF (N, %)	37 (84.1)
HR (beats/min)	138 ± 19
SBP (mmHg)	116 ± 21
DBP (mmHg)	70 ± 15
LVEF (%)	42.7 ± 16.5
LVDd (mm)	49.3 ± 12.8
LVDs (mm)	38.6 ± 11.1
LAD (mm)	42.1 ± 6.4
Ischemic heart disease (N, %)	16 (36.4)

Hb: hemoglobin; BNP: brain natriuretic peptide; AF: atrial fibrillation; HR: heart rate; SBP: systolic blood pressure; DBP: diastolic blood pressure; LVEF: left ventricular ejection fraction; LVDd: left ventricular diastolic diameter; LVDs: left ventricular systolic diameter; LAD: left atrium diameter; HbA1c: glycated hemoglobin.

**Table 2:** Differences in the characteristics between responders (n = 24) and non-responders (n = 20) to landiolol.

	Responders (n = 24)	Non-Responders (n = 20)	*p* Value
Age (years old)	77.8 ± 10.8	79.6 ± 11.6	0.479
Male (N, %)	14 (58.3)	13 (65)	0.76
Weight (kg)	57.9 ± 13.8	55.4 ± 11.8	0.61
Hb (g/dL)	12.0 ± 2.6	12.9 ± 3.2	0.2
BNP (pg/mL)	695 ± 469	635 ± 421	0.8
HbA1c (%)	6.0 ± 1.0	6.5 ± 1.2	0.01
Creatinine (mg/dL)	1.3 ± 1.2	1.5 ± 0.9	0.05
AF (N, %)	24 (100)	13 (65)	0.002
Pre HR (bpm)	137 ± 20	140 ± 18	0.48
Pre SBP (mmHg)	115 ± 21	117 ± 22	0.85
Pre DBP (mmHg)	71 ± 14	68 ± 17	0.58
Post HR (bpm)	95 ± 14	133 ± 17	<0.01
Post SBP (mmHg)	103 ± 20	113 ± 20	0.15
Post DBP (mmHg)	61 ± 9	67 ± 15	0.25
LVEF (%)	42.0 ± 17.1	43.6 ± 16.1	0.75
LVDd (mm)	50.1 ± 14.1	48.2 ± 11.1	0.5
LVDs (mm)	38.6 ± 10.8	38.7 ± 12.0	0.83
LAD (mm)	41.9 ± 5.7	42.5 ± 7.4	0.83
Ischemic heart disease (N,%)	9 (37.5)	7 (35)	0.916
The dosage of landiolol (μg/kg/min)	3.7 ± 1.5	4.2 ± 2.6	0.972

Hb: hemoglobin; BNP: brain natriuretic peptide; HbA1c: glycated hemoglobin; AF: atrial fibrillation; HR: heart rate; SBP: systolic blood pressure; DBP: diastolic blood pressure; LVEF: left ventricular ejection fraction; LVDd: left ventricular diastolic diameter; LVDs: left ventricular systolic diameter; LAD: left atrium diameter; n: number, number ± (standard deviation).

**Table 3:** Differences in the characteristics between patients with AT and those with AF.

	AT Group (n = 7)	AF Group (n = 37)	*p*-Value
Age (years)	75.3 ± 17.1	79.2 ± 9.8	0.86
Hb (g/dL)	12.1 ± 2.1	12.4 ± 3.0	0.76
BNP (pg/mL)	537 ± 411	694 ± 448	0.506
HbA1c (%)	6.3 ± 1.1	6.2 ± 1.1	0.54
Creatinine (mg/dL)	1.3 ± 0.4	1.4 ± 1.2	0.54
LVEF (%)	44.1 ± 15.3	42.4 ± 16.9	0.78
Pre HR (bpm)	146 ± 23	137 ± 18	0.32
Post HR (bpm)	143 ± 22	107 ± 20	<0.01
Spontaneous conversion to sinus rhythm (n, %)	0 (0)	15 (44.1)	<0.01
Landiolol dosage (μg/kg/min)	4.8 ± 1.5	3.8 ± 1.6	0.97

Hb: hemoglobin; BNP: brain natriuretic peptide; AF: atrial fibrillation; LVEF: left ventricular ejection fraction; HR: heart rate; HbA1c: glycated hemoglobin.

## Data Availability

The original contributions presented in the study are included in the article material, further inquiries can be directed to the corresponding authors.
